# A Synthetic Curcuminoid Analog, (2*E*,6*E*)-2,6-bis(2-(trifluoromethyl)benzylidene)cyclohexanone, Ameliorates Impaired Wound Healing in Streptozotocin-Induced Diabetic Mice by Increasing miR-146a

**DOI:** 10.3390/molecules25040920

**Published:** 2020-02-19

**Authors:** Jingjuan Huang, Jia Fu, Bing Liu, Rui Wang, Tianhui You

**Affiliations:** 1 School of Nursing, Guangdong Pharmaceutical University, Guangzhou 510006, China; 18825077178@163.com (J.H.); fjsxyd1012@163.com (J.F.); 2 Guangzhou Key Laboratory of Construction and Application of New Drug Screening Model Systems, Guangdong Pharmaceutical University, Guangzhou 510006, China; liubing520@gdpu.edu.cn; 3 Key Laboratory of New Drug Discovery and Evaluation of Ordinary Universities of Guangdong Province, Guangdong Pharmaceutical University, Guangzhou 510006, China; 4 School of Pharmacy, Guangdong Pharmaceutical University, Guangzhou 510006, China; 5 Guangdong Key Laboratory of Pharmaceutical Bioactive Substances, Guangdong Pharmaceutical University, Guangzhou 510006, China

**Keywords:** curcuminoid, synthetic analog, wound healing, miR-146a, inflammation, NF-κB

## Abstract

The impairment in diabetic wound healing represents a significant clinical problem, with no efficient targeted treatments for these wound disorders. Curcumin is well confirmed to improve diabetic wound healing, however, its low bioavailability and poor solubility severely limit its clinical application. This study aims to provide the pharmacological basis for the use of (2*E*,6*E*)-2,6-bis(2-(trifluoromethyl)benzylidene)cyclohexanone (C66). The results showed that topically applied C66 improved cutaneous wound healing in vivo. Further studies showed that C66 treatment increased the level of microRNA-146a (miR-146a) in the wounds in streptozotocin (STZ)-induced diabetic mice, downregulated the expression of interleukin-1 receptor-associated kinase 1 (IRAK1) and phosphorylated nuclear factor-κB (NF-κB) p65 subunit (p-p65) (both *p* < 0.05), and suppressed the mRNA expression of inflammation-related cytokines, tumor necrosis factor-α (TNF-α), interleukin-8 (IL-8), and interleukin-6 (IL-6). The in vitro data obtained in human umbilical vein endothelial cells (HUVECs) showed that C66 could reverse high glucose (HG)-induced NF-κB activation due to upregulation of miR-146a expression, which matched the in vivo findings. In conclusion, the present study indicates that C66 exerts anti-inflammation activity and accelerates skin wound healing of diabetic mice, probably via increasing miR-146a and inhibiting the NF-κB-mediated inflammation pathway. Therefore, C66 may be a promising alternative for the treatment of diabetic wounds.

## 1. Introduction

The impairment in diabetic wound healing represents a significant clinical problem [1], however, to date, efficient targeted treatment for these wound disorders remains lacking. During the wound healing process, the initial post-wounding inflammatory phase is critical for normal healing, but if persistent, results in a chronically inflamed wound that is difficult to heal [2]. 

Curcumin (1,7-bis-(4-hydroxy-3-methoxyphenyl)-1,6-heptadiene-3,5-dione), a polyphenol extracted from the plant curcuma longa with anti-inflammatory and free-radical scavenging function, has been proven to be an effective agent against wound [3,4,5], but the exact mechanisms remain unclear. However, the clinical application of curcumin is limited due to its poor water solubility and low bioavailability [6]. A novel curcumin analogue (2*E*,6*E*)-2,6-bis(2-(trifluoromethyl)benzylidene) cyclohexanone (C66) is one of the derivatives of curcumin [7,8]. Systemic administration of C66 has been shown to exhibit anti-inflammatory properties in several disease conditions with higher stability and lower dosage [9,10].

MicroRNAs (miRs) are short noncoding RNAs that are approximately 21–25 nucleotides in length and believed to regulate up to 50% of all protein-coding genes [11]. Several miRNAs have been found in skin tissue and are thought to play an indispensable role in the regulation of innate and adaptive immune responses [12,13]. MiR-146a, with its adaptor genes (TNF receptor-associated factor 6 (TRAF6) and interleukin-1 receptor associated kinase 1 (IRAK1), stimulates the nuclear factor kappa B (NF-κB) signaling pathway and correlates with inflammation in diabetic rat hippocampus [14] as well as diabetic wound healing [15]. Interestingly, a difluorinated analogue of curcumin upregulates expression of the miR-146a family [16]. However, whether C66 also regulates miR-146a and improves diabetic wound healing remain unidentified. Therefore, in this study, we sought to uncover the potential role of curcumin analogue C66 in diabetic wound healing and the underlying mechanisms.

## 2. Results

### 2.1. Effect of C66 on Blood Glucose Level and Body Weight in Diabetic Mice

**Figure 1:**
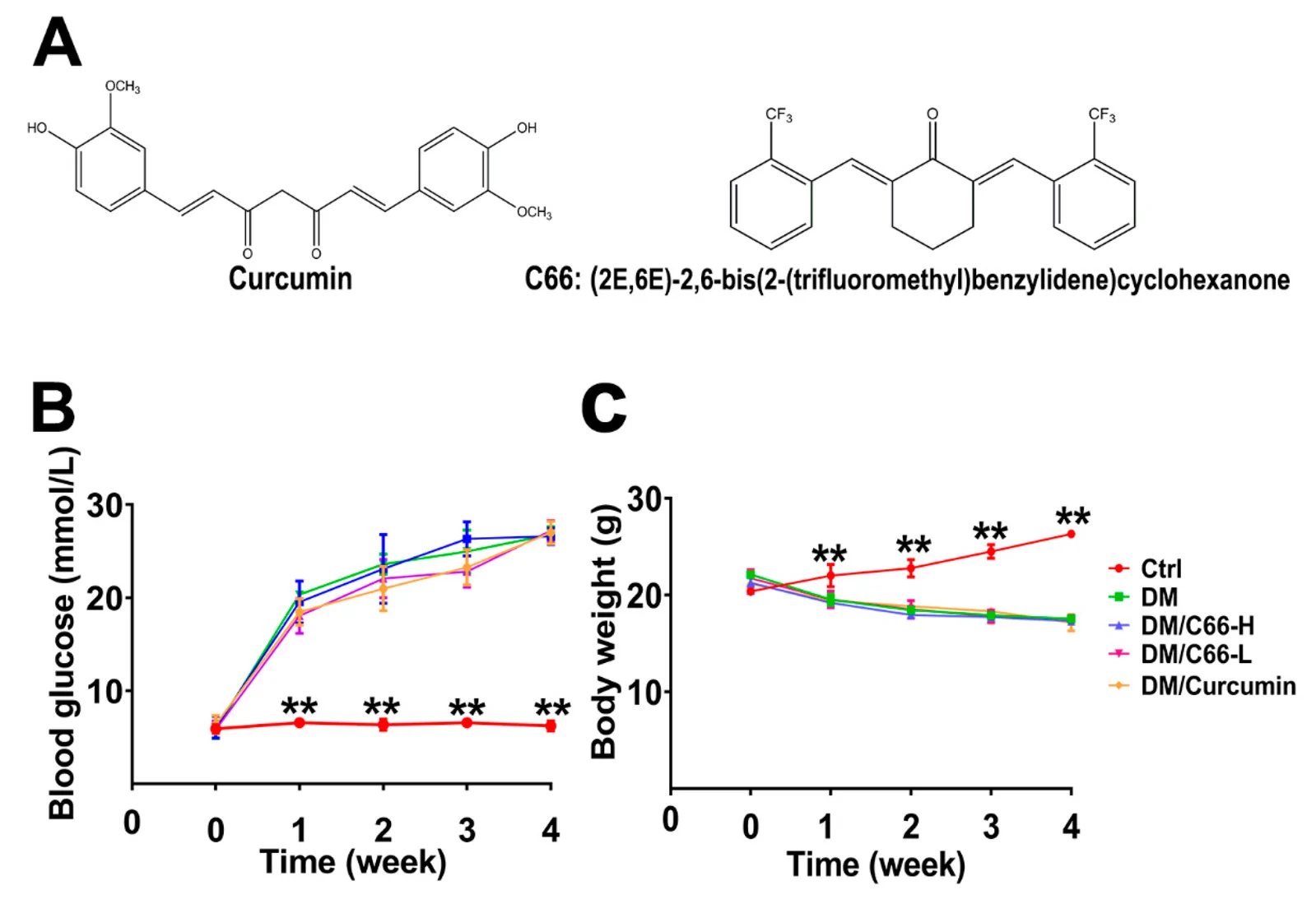
Establishment of streptozotocin (STZ)-induced diabetic mice. The mice with blood glucose greater than 16.7 mmol/L were defined as diabetic mice. (**A**) Chemical structures of curcumin and C66. Blood glucose levels (**B**) were monitored during the study period. Body weight (**C**) was significantly decreased in the diabetic group and C66 treatment group compared with the control group. The data are expressed as the means ± S.D. (*n* = 6 per group, ** *p* < 0.01 compared to the control value). DM and Ctrl are short for the diabetic and control group, respectively.

The structures of curcumin and its analog C66 with the molecular weight of 410 g/mol used in this study are shown in Figure 1A. During the treatment period, the blood glucose levels of mice in the diabetic group (DM) were significantly higher than those of mice in the control group (Ctrl), indicating the success of the development of diabetic mice models (Figure 1B). Curcumin or C66 treatment had little effect on blood glucose.

Additionally, the body weight of the mice was also detected. The body weights of mice in the diabetic group and treatment groups (curcumin or C66) were significantly lower than those of mice in the control group, whereas no significant difference was observed between the diabetic group and treatment groups (curcumin or C66) (Figure 1C), indicating that both curcumin and C66 had no effect on mice weight during the experimental period.

### 2.2. C66 Accelerates Skin Wound Healing

**Figure 2:**
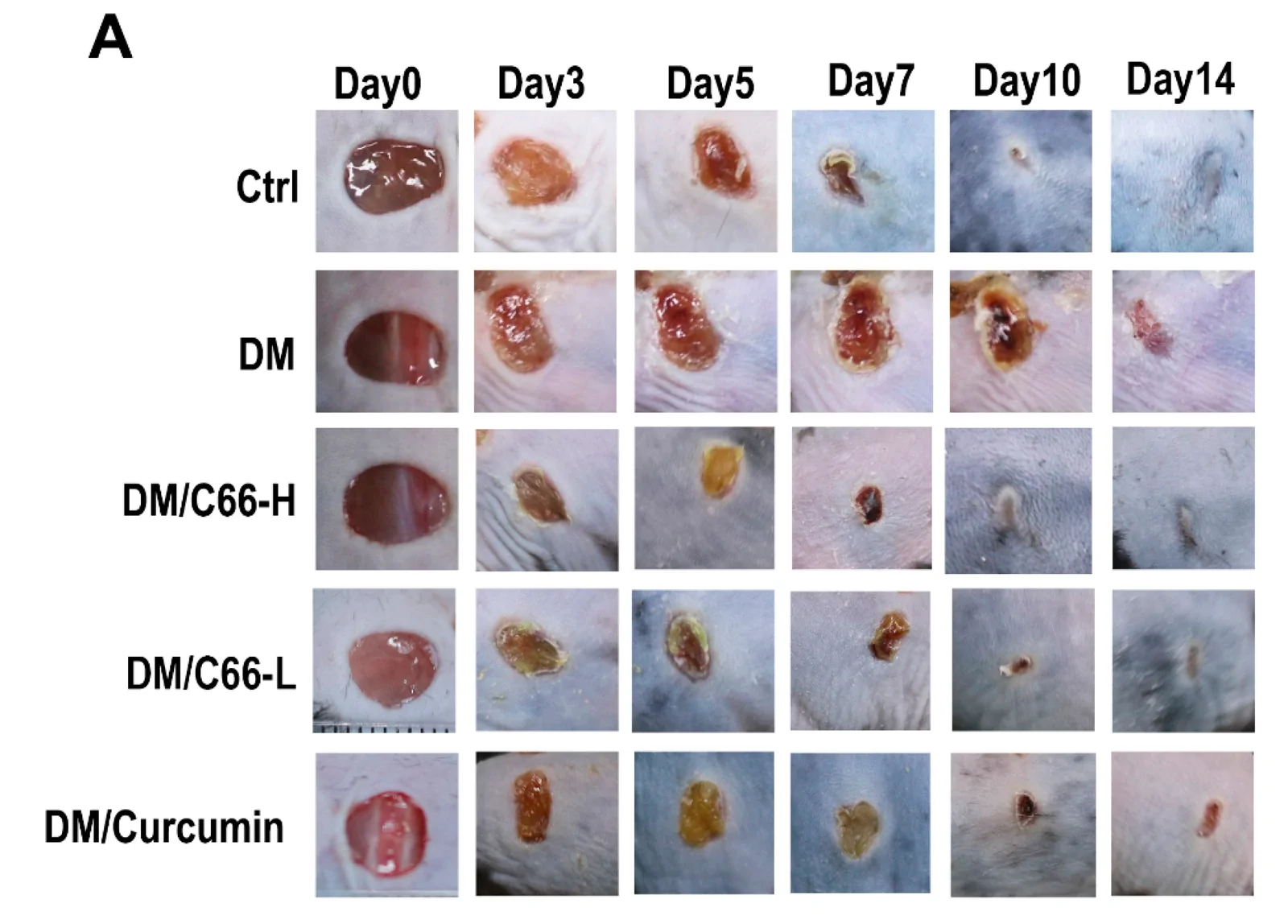

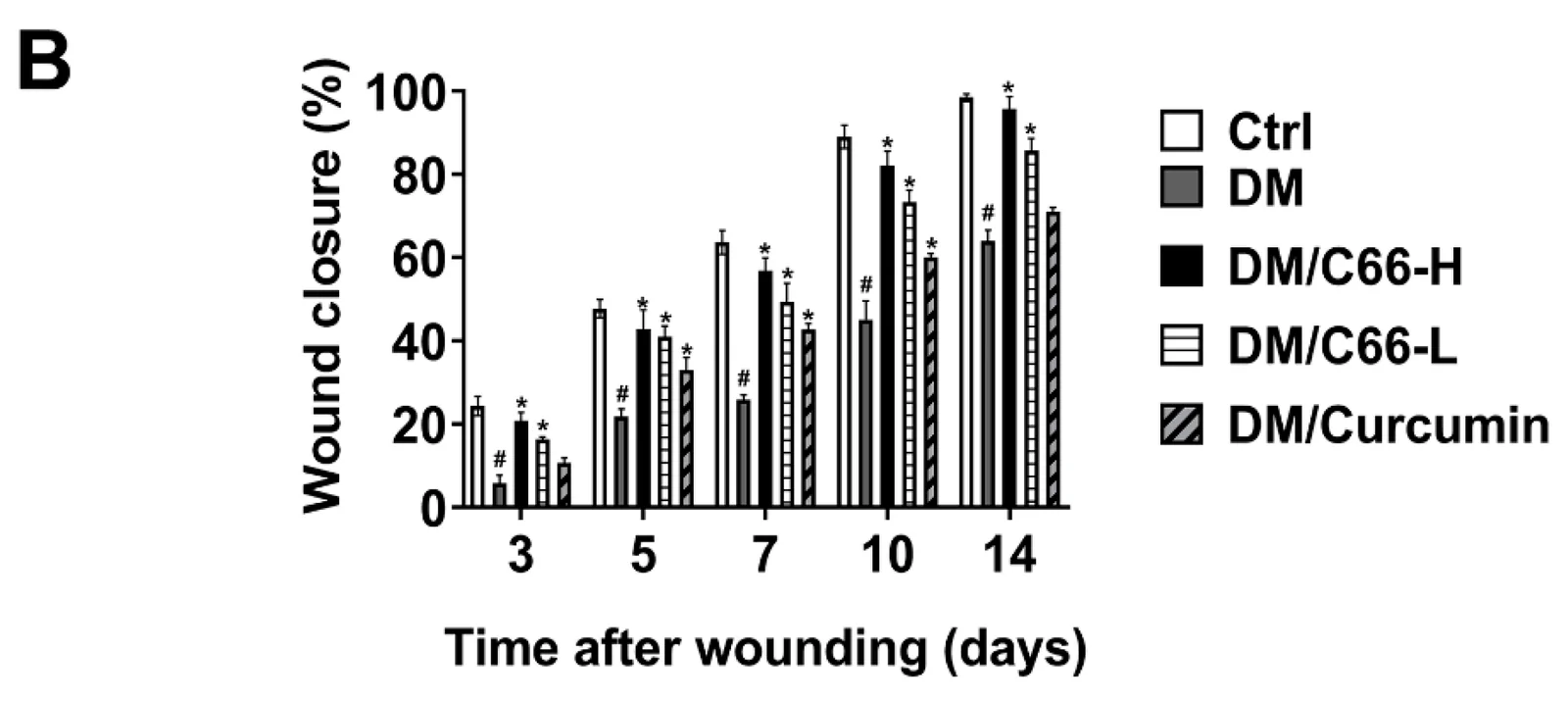
C66 accelerates diabetic wound healing. (**A**) Six millimeter diameter wounds were created by punch biopsy, and the closure of the wound area was measured by digital camera until day 14. (**B**) Percentage of wound closure (means ± S.D.). Healing of diabetic wounds significantly delayed compared with normal wounds. The C66 group began to improve diabetic wound closure on day 3. At the end of the observation period (14 days), the C66 treatment group exhibited improved wound healing, compared with the diabetic group. Data were represented as means ± S.D. * *p* < 0.05 compared to the diabetes group value, # *p* < 0.05 compared to the control value. DM and Ctrl are short for the diabetic and control group, respectively.

Figure 2A shows the representative ulceration images for each group. Figure 2B shows the wound closure of ulceration at the indicated time points. By the end of the observation period (day 14 after wounding), normal wounds and C66 (both low dose and high dose)-treated wounds were completely healed, while most of the diabetic wounds remained open with a low average closure rate of approximately 64.0%. C66 (both low dose and high dose) significantly improved diabetic wound closure and increased the healing rate of diabetic wound at least by 21.7% (85.7% versus 64.0%, *p* < 0.05). Furthermore, curcumin also improved the diabetic wound closure, but the healing rate was obviously lower than that of the C66 treatment group.

### 2.3. Pathologic Study on Diabetic Wound Healing by C66

**Figure 3:**
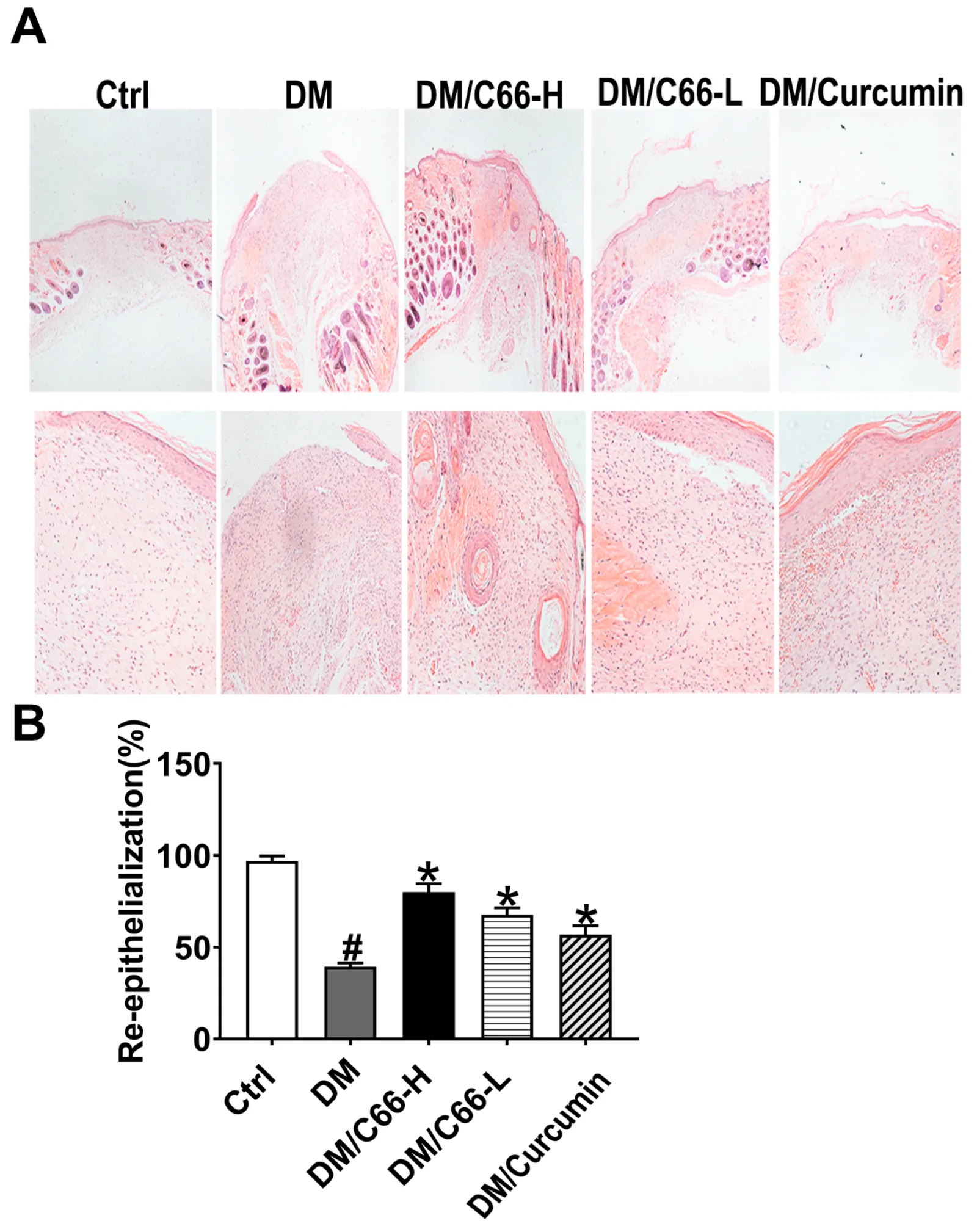
Reepithelialization of skin wounds was accelerated in the C66-treated mice. (**A**) Histological reepithelialization of skin wound in different groups was measured at day 14 after injury (H&E staining, ×100). (**B**) The ratio of reepithelialization was evaluated in different groups. Data are presented as the means ± S.D (*n* = 6 in each group). * *p* < 0.05 compared to the diabetes group value, # *p* < 0.05 compared to the control value. DM and Ctrl are short for the diabetic and control group, respectively.

Re-epithelialization was measured at day 14 after wounding as examined by the histomorphometric analysis of sections stained with hematoxylin-eosin (H&E) (Figure 3A). As shown in Figure 3, at the end of the observation period, wounds were not fully re-epithelialized in the diabetic group with the rate of re-epithelialization of 40% at day 14 after injury, while the wounds got close to fully re-epithelialized in the control group. With C66 and curcumin supplementation, the epithelia were significantly longer when compared with those of the diabetic group, and the C66-treated mice showed a significantly accelerated re-epithelialization compared with curcumin-treated mice (Figure 3B). These observations suggest that C66 promotes re-epithelialization in diabetic wounds, with the efficacy superior to curcumin.

**Figure 4:**
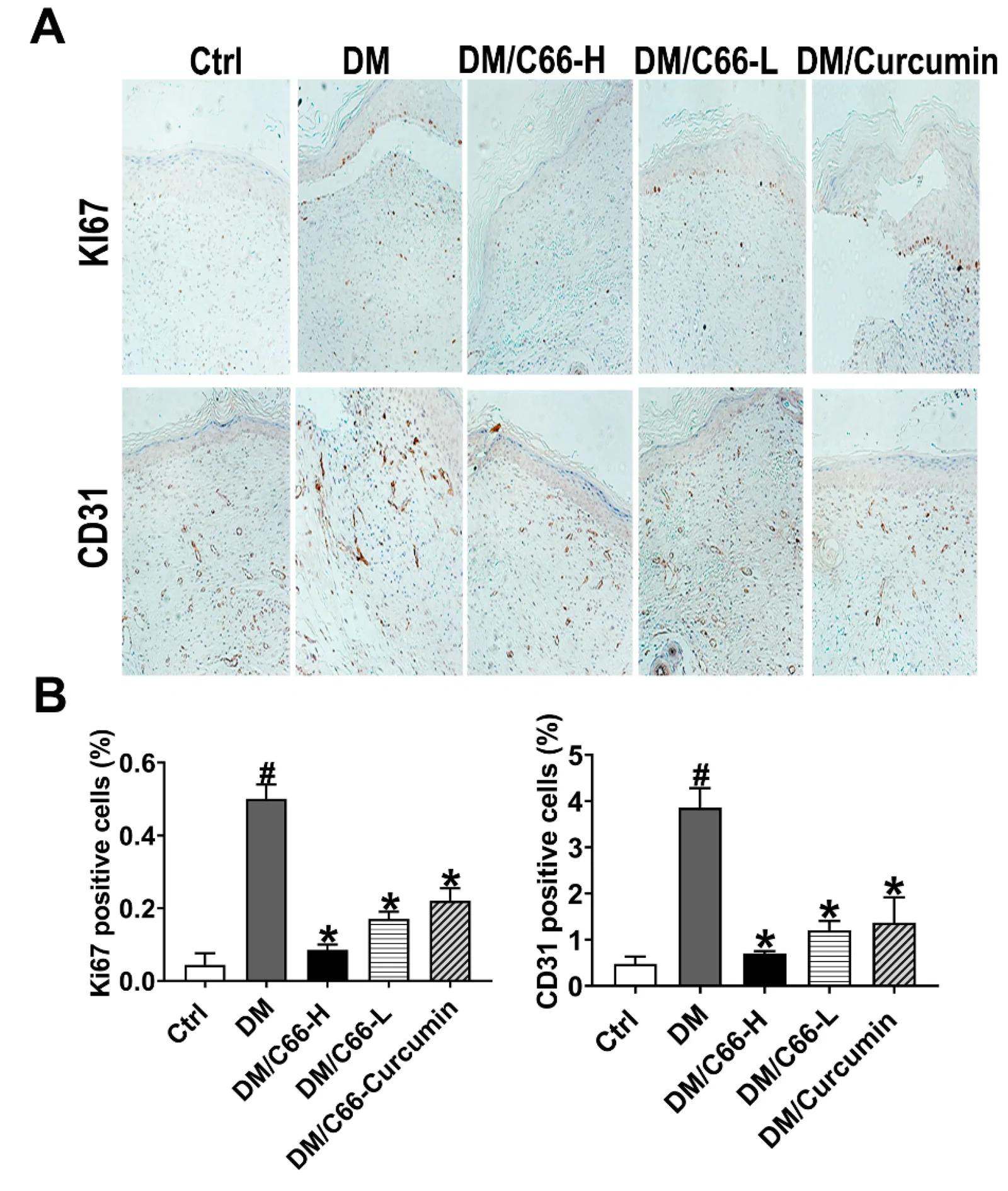
C66 stimulated cellular proliferation, and augmented neovascularization on the wound bed. (**A**) Cellular proliferation in the wound tissues was detected with the immunostaining of Ki67. The neovascularization of wounds was detected by immunostaining of endothelial marker CD31. (**B**) Scores of Ki67 and CD31 staining; n = 6 for each group. Data are represented as means ± S.D. * *p* < 0.05 compared to the diabetes group value, # *p* < 0.05 compared to the control value. DM and Ctrl are short for the diabetic and control group, respectively.

Another finding that supported the delayed wound healing in the diabetic group wound was the ratio of proliferative cells in the basal layer of the new epithelium (Figure 4A,B). On day 14, many Ki-67-positive cells were still found in the basal layer of skin from diabetic wounds, however, there was a significant decline of Ki-67-positive cells in the healed skin of the C66 (both low dose and high dose) and curcumin treatment groups, with the ratio of proliferative cells of C66 (high dose) group comparable to that of the control group. 

Neovascularization is an essential event in the healing of wounds. We evaluated the neovascularization by immunostaining of endothelial marker CD31. On day 14, neovascularization of diabetic wounds was significantly decreased in the C66 (high dose)-treated group, compared with the diabetic group (0.70 ± 0.06 versus 3.86 ± 0.43, *p* < 0.05) (Figure 4A,B).

### 2.4. C66 Attenuates NF-κB Activity via miR-146a Targeting IRAK1

**Figure 5:**
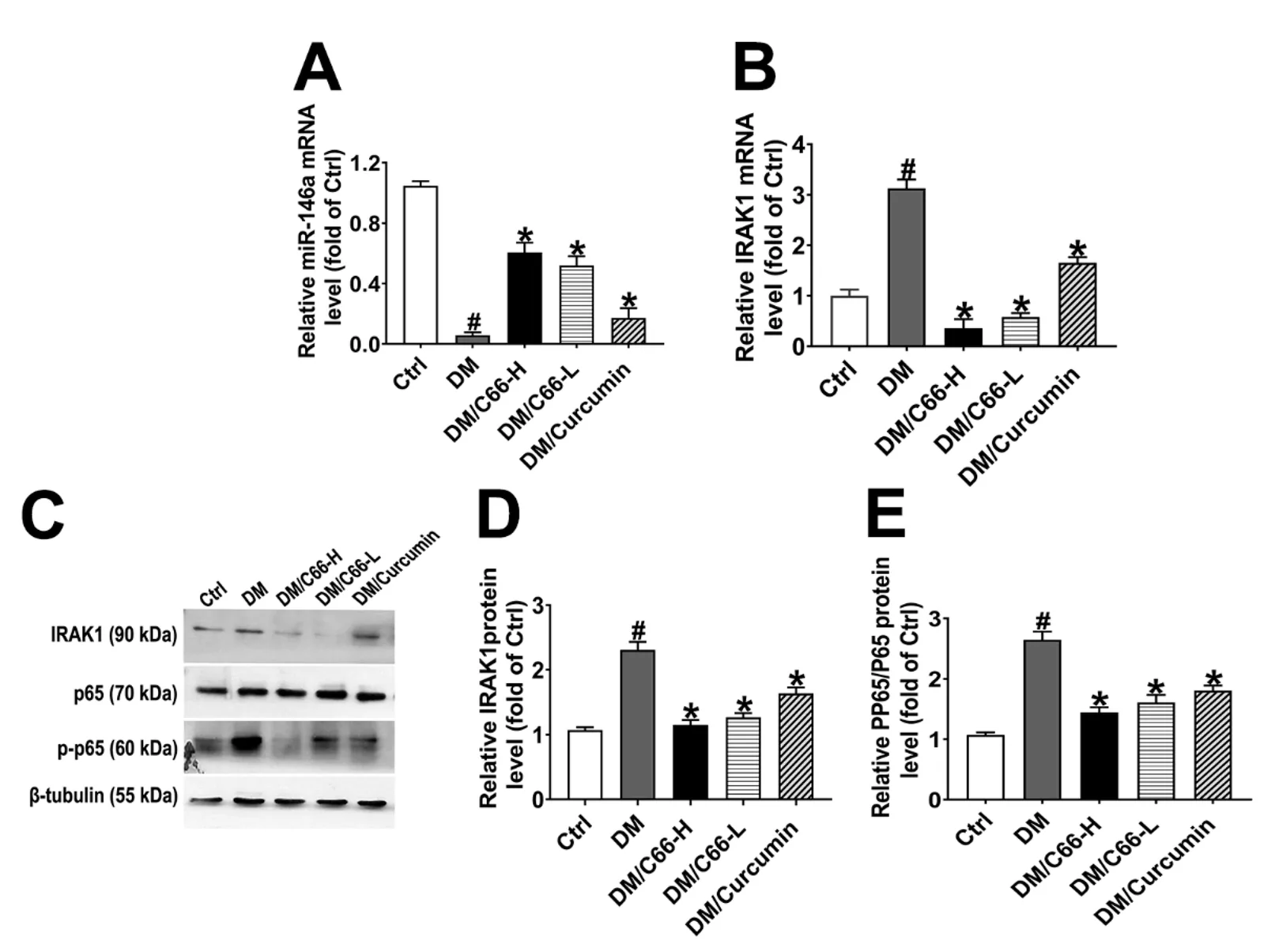
C66 increased miR-146a, leading to downregulation of IRAK1 and the effects of curcumin and C66 on the wound expression of pp65/p65 and IRAK1. RNA levels for miR-146a (**A**) and IRAK1 (**B**) were determined by quantitative real-time polymerase chain reaction (PCR). Expression of IRAK1, β-tubulin, p65, and p-p65 was measured by western blot analysis (**C**). Graphs showed the semiquantitative analysis of protein levels (**D**,**E**). Data are normalized by the control and presented as means ± S.D. (*n* = 6). * *p* < 0.05 compared to the diabetes group value, # *p* < 0.05 compared to the control value. DM and Ctrl are short for diabetic and control group, respectively.

The next study was to address whether C66 upregulates miR-146a and suppresses the NF-κB signaling pathway. miR-146a was significantly decreased in diabetic wounds, while C66 increased miR-146a in diabetic wounds (Figure 5A). C66 decreased diabetes-induced high levels of IRAK1 mRNA (Figure 5B). The elevated expression of IRAK1 and phosphorylated NF-κB p65 subunit (p-p65) proteins induced by hyperglycemia was attenuated by C66 (Figure 5C–E). These results support that C66 increases miR-146a expression and inhibits the NF-κB signaling pathway in diabetic wounds. 

### 2.5. MiR-146a Mediates the Inhibitory Effect of C66 on High Glucose-Induced NF-κB Activation through Targeting IRAK1

**Figure 6:**
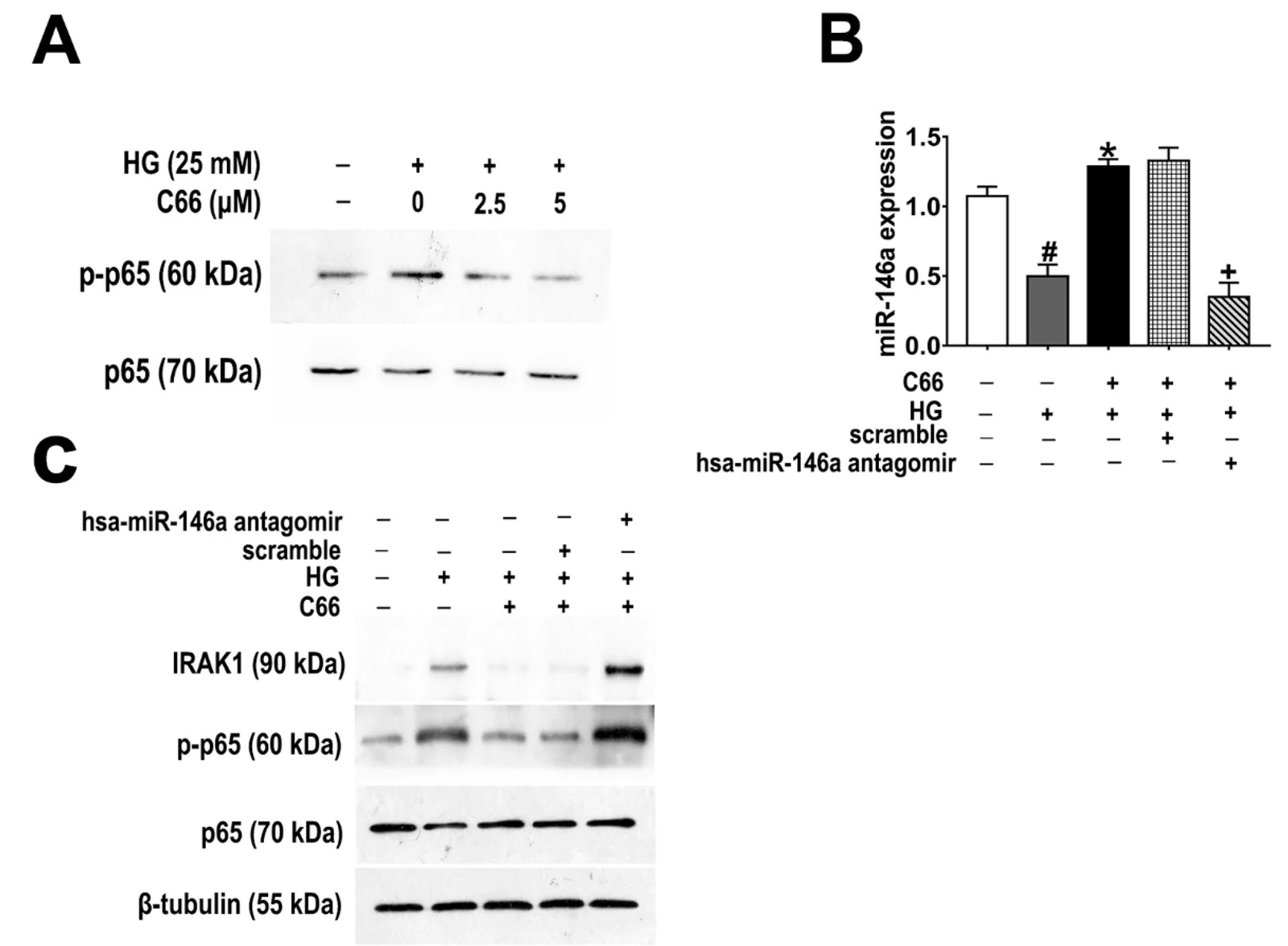
C66 inhibited the high glucose (HG)-induced phosphorylation of NF-κB signaling and the miR-146a antagomir completely abolished the effect of C66 on the NF-κB signaling pathway in HUVECs cells. (**A**) C66 dose-dependently reduced HG-induced protein expression of p-p65. (**B**) The effects of C66, HG, hsa-miR-146a antagomir, and their combinations were compared on RNA levels of miR-146a. Bar graphs represent the mean ± SD of three independent experiments. Results are expressed as fold change relative to untreated control cells. # *p* < 0.05 vs. control cells; * *p* < 0.05 vs. hyperglycemic conditions alone; + *p* < 0.05 vs. HG/C66/scramble; (**C**) Knockdown of miR-146a in HUVECs by hsa-miR-146a antagomir transfection reversed the inhibitory effect of C66 on HG-induced NF-κB activation.

As Figure 6A shows, C66 inhibited high glucose (HG) (25 mM)-induced expression of phospho-p65 in a dose-dependent manner (0–5 µM) after 24-hour treatment in human umbilical vein endothelial cells (HUVECs). HG-induced NF-κB activation is mediated by IRAK1, one-key adaptor kinase downstream of the Toll-like receptor (TLR) superfamily through phosphorylation and proteasome-mediated degradation of the IκB protein [17]. Next, we sought to determine whether C66 inhibited HG-stimulated NF-κB signaling via miR-146a upregulation. As expected, HG (25 mM) treatment led to a substantial reduction in the expression of miR-146a, which was rescued by C66 administration (Figure 6B). Furthermore, pre-transfection with hsa-miR-146a antagomir completely abolished the upregulation of miR-146a by C66 in HG-incubated HUVECs (Figure 6B). As shown in Figure 6C, C66 (5 µM) could substantially block the enhancement effect of HG on IRAK1 and p-p65 expression in HUVECs, however, these effects were reversed by anti-miR-146a treatment. Taken together, these results clearly indicate that C66 can reverse HG-induced NF-κB activation due to the stimulation of miR-146a expression in HUVECs. 

### 2.6. C66 Decreases Pro-Inflammatory Factors

**Figure 7:**
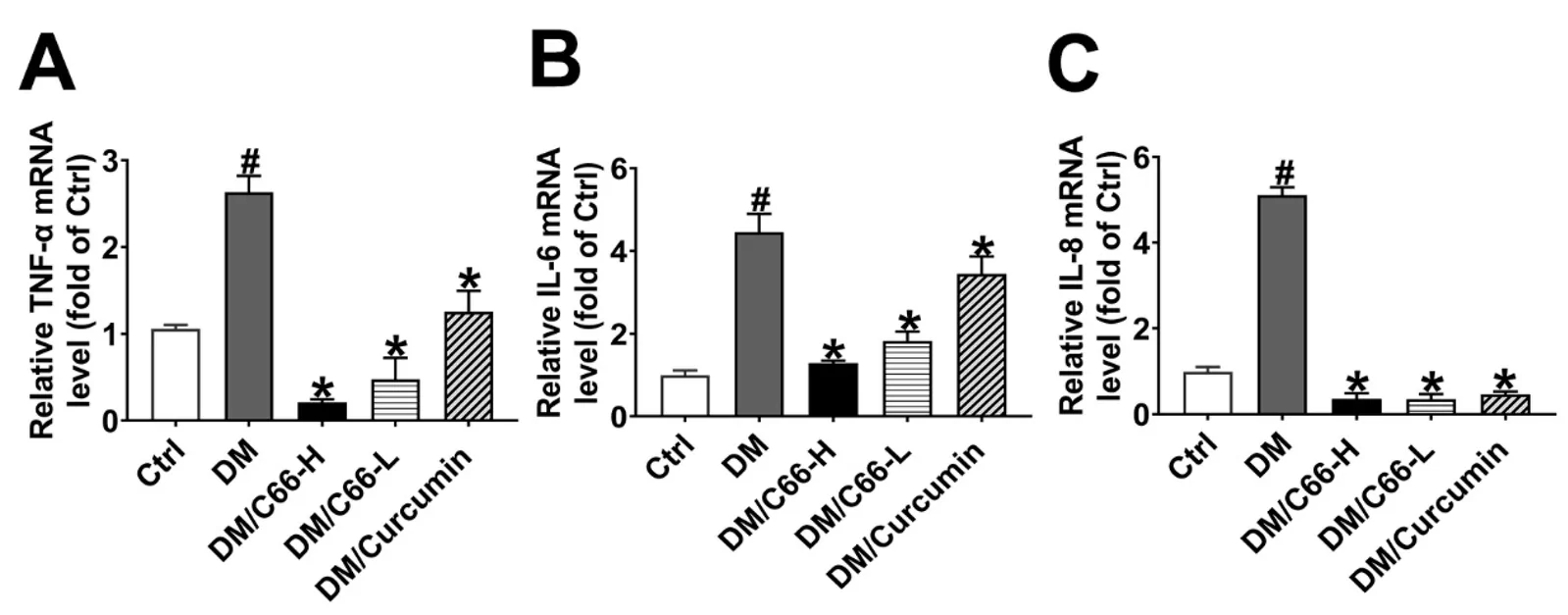
C66 significantly downregulated pro-inflammatory cytokines. Real-time quantitative RT-PCR analysis of mRNA for TNF-α (**A**), IL-6 (**B**), and IL-8 (**C**) in the 14-day wounds of different groups. The expression levels of pro-inflammatory cytokines were significantly downregulated in the C66 treatment group compared with the diabetes group (*p* < 0.05). * *p* < 0.05 compared to the diabetes group value, # *p* < 0.05 compared to the control value.

Further measurement of pro-inflammatory cytokines demonstrated that streptozotocin (STZ) treatment increased tumor necrosis factor-alpha (TNF-α), interleukin (IL)-6 (IL-6), and interleukin (IL)-8 (IL-8) mRNA levels in the diabetic wounds (Figure 7A–C). Curcumin treatment markedly reduced the mRNA expression of those cytokines. Compared with curcumin, C66 administration had more pronounced inhibition effects on STZ-induced pro-inflammatory cytokine (TNF-α, IL-6, and IL-8) expression. These results strongly support that C66 can inhibit inflammatory reactions in the diabetic wounds.

## 3. Discussion

Chronic non-healing ulcers, commonly found in diabetic patients, represent a significant source of morbidity and expense to both patients and the healthcare system. Persistent inflammation and delayed epithelialization contribute to the stalled healing in these ulcers. Herein, we have illustrated the potential use of a curcumin analog in an impaired wound healing model using a topical treatment of C66 that decreases inflammatory cytokines. 

Curcumin is a regulator of epigenetic events including miRNAs [18], among which miR-146a has been demonstrated to play a key role in the pathogenesis of diabetic non-healing wounds. However, certain pharmacokinetic defects such as low bioavailability and poor chemical stability significantly limit the clinical application of curcumin. Systemic administration of C66 has been shown to exhibit anti-inflammatory properties in several disease conditions with higher stability and lower dosage [9,10]. However, the potential for local modulation of the inflammatory environment in a chronic wound by topical application of C66 has not been explored yet. Our results clearly demonstrate that C66 preserves curcumin’s function and is more potent in anti-inflammation.

One of the novel findings of our study is that C66 treatment significantly upregulated miR-146a expression in diabetic wounds. Such upregulation was accompanied by a dramatic downregulation of its target genes, IRAK1, and the levels of TNF-α, IL-6, and IL-8, which are key mediators of inflammation and well known as downstream inflammatory genes of the NF-κB pathway [19,20]. Therefore, it is reasonable to postulate that the inhibition of the inflammatory NF-κB pathway is involved in the process of wound healing by C66. Furthermore, the data also provide evidence that C66 enhances wound repair by altering miR-146a expression and attenuating the NF-κB pathway-mediated inflammatory response. 

Due to the limited tissue available for harvest from the wound, this study provides only the gene expression data of the proposed signaling pathways at the wound site. However, the present study demonstrates the first time that C66 ameliorates impaired diabetic wound healing by suppressing the NF-κB-regulated inflammatory genes through the upregulation of miR-146a. In addition, our results suggest that miR-146a may also represent a potential useful marker of inflammation as well as a potential therapeutic target for the modification of the diabetic wound-healing response.

## 4. Materials and Methods 

### 4.1. Drugs and Chemicals

Compound C66 was synthesized and characterized by Boshi Environmental Protection Project Co. Ltd. A high-performance liquid chromatography method was used to determine its purity (98.67%). The structure of C66 was shown in Figure 1. STZ was purchased from Sigma-Aldrich (St. Louis, MO, USA). Curcumin and C66 were suspended in 0.5% sodium carboxymethylcellulose (CMC-Na) to improve its topical application. 

### 4.2. Animals and Ethics Statement

Adult male C57BL/6 mice of eight weeks old with body weights of 20 to 22 g were obtained from the Center of Experimental Animal of Guangdong Province (permit number: SCXK (Yue) 20080002) and used to establish a mouse model for diabetes mellitus. All mice were lodged in individual cages in a temperature- and humidity-controlled room (22 ± 1 °C and 50 ± 1% humidity) with a 12-hour light cycle in the animal facility of the Animal Unit of Guangdong Pharmaceutical University. Water and mouse standard diet were used. Protocols for animal studies were conducted according to the National Institutes of Health (NIH) Guide for the Care and Use of Laboratory Animals and approved by the Guangdong Pharmaceutical University Animal Care and Use Committee and all efforts were made to minimize the suffering of animals. 

### 4.3. Induction of DM and Experimental Design

Diabetes was induced in mice with STZ injected intraperitoneally on five consecutive days at a dose of 50 mg/kg (dissolved in 0.1 M sodium citrate, pH 4.3–4.5) [21]. Nondiabetic mice were injected with only a saline vehicle. One week after the last injection of STZ, fasting glucose levels (4 h fast) were measured and mice with fasting blood glucose levels higher than 16.7 mM (300 mg/dL) were considered as diabetic. Six mice were excluded from the study after the confirmation of success of diabetic models because of low blood glucose levels. Thirty mice were randomly divided into five groups as follows: (1) diabetic mellitus group (DM): diabetic mice were topically given 0.5% CMC-Na for 14 days; (2) low-dose C66 group (DM/C66-L): diabetic mice were topically given 200 μL of C66 daily at 10 μM for 14 days; (3) high-dose C66 group (DM/C66-H): diabetic mice were topically given 200 μL of C66 daily at 20 µM for 14 days; and (4) positive control group (DM/curcumin): diabetic mice received 200 μL of curcumin daily at 20 μM for 14 days. Six nondiabetic mice who received a 0.5% CMC-Na vehicle for 14 days were used as the control group.

### 4.4. Wound Biopsy and Measurement of Wound Closure

After two weeks of STZ induction, a model for diabetic wound was created as follows: mice were anesthetized with 1% pentobarbital (30 mg/kg), and the hair on their back was shaved. Circular, full-thickness skin excisions of 6 mm in diameter in the middle of the back at each side of the spine were aseptically generated. After recovery from the anesthesia, animals were housed individually in properly disinfected cages. Wounds in individual mice were photographed digitally on days 0, 3, 5, 7, and 10 until the end (day 14). A digital camera (EOSD80, Cannon, Japan) was employed to take pictures, and the ulcer area was analyzed by Image-Pro Plus 4.5 software. The wound closure area was calculated using Wilson’s formulas as a percentage of their original area, calculated as

[(open area at postoperative day 0 minus open area at postoperative day X)/(wound area at postoperative day 0)] × 100.(1)

### 4.5. Histological Analysis of Wound Healing

Skin tissues were dissected quickly on ice. Parts of them were immediately fixed for histological analysis, and others were stored in −80 °C for biochemical and molecular analysis. Wound specimens were harvested and fixed in 4% formaldehyde buffered with phosphate buffer saline (PBS) (pH 7.2), and then embedded with paraffin. Five-micrometer thick sections were stained with H&E or modified Masson’s trichrome, according to the manufacturer’s protocol (American Master-Tech, Lodi, CA, USA). We analyzed the degree of reepithelialization as described previously [22]. Briefly, the percentage of re-epithelialization (distance traversed by epithelium over wound from wound edge/distance between wound edge) was calculated for two sections per wound and was averaged over sections to provide a representative value for each wound.

### 4.6. Immunohistochemistry for Ki-67 and CD31

The sections were incubated with the primary antibodies against CD31 (1:50, Abcam, Cambridge, MA, USA) and Ki-67 (1:50, Abcam, USA). To calculate the percentage of Ki-67-positive cells, analyses were performed by counting the total number of basal cells and cells expressing nuclear Ki-67.

### 4.7. Detection of miRNA and mRNA Expression 

Gene expression was determined by quantitative real-time polymerase chain reaction (qRT-PCR). Total RNA including miRNAs was extracted from skin tissues (30 mg) and cell cultures using Trizol reagent (Invitrogen, Shanghai, China) and was dissolved in RNase-free water, according to the manufacturer’s instructions. Reverse transcription was carried out using a RevertAid First Strand cDNA Synthesis Kit (Thermo Scientific, Rockford, IL, USA). Total RNA (1 μg) was used as a template for single strand cDNA synthesis. The resultant cDNA was amplified using a SYBR Green qPCR kit (Toyobo, Osaka, Japan) and the subsequent quantification using an ABI PRISM 7500 sequence detection system (Applied Biosystems, Life Technologies, Grand Island, NY, USA). The primers of genes described in Table 1 were synthesized from Invitrogen (Invitrogen, Shanghai, China). The method to quantify miRNAs was performed by stem-loop RT-PCR. MiRNAs and reverse primers were put at 65 °C for 5 min to form a highly target-specific stem-loop structure. Then, reverse transcriptase, RNase inhibitor, dNTPs, and buffer were added for reverse transcription, with small nuclear RNA U6 as the normalization control. For mRNAs analysis, the amount of each gene was determined and normalized to the amount of glyceraldehyde-3-phosphate dehydrogenase (GAPDH) using 7500 system SDS software. Relative differences of gene expression between the different groups were calculated using the formula 2^−△△Ct^.

### 4.8. Western Blot Analysis

The expression of related proteins was detected by western blot analysis. Tissues and cells were homogenized in radio immunoprecipitation assay buffer in the presence of protease inhibitors. Protein (20 μg) from the homogenates was fractionated on sodium dodecyl sulfate polyacrylamide gel electrophoresis (SDS-PAGE) gel and transferred to nitrocellulose membranes (MSI, Westboro, MA, USA). The membrane was first probed with primary antibodies as follows: IRAK1 (1:1000, GeneTex, Irvine, CA, USA), p65 (1:1000, GeneTex, USA), phospho-p65 (1:1000, GeneTex, USA), and β-Tubulin (1:1000, GeneTex, USA). The secondary antibody were Goat anti-mouse or Goat anti-rabbit IgG (Proteintech, Chicago, IL, USA, SA00001-1 and SA00001-2), respectively. After that, the immunoreactive bands were detected with enhanced chemiluminescent (ECL) reagent (Beyotime, Shanghai, China). The data were quantified using Image Studio Lite ver. 5.2 software.

### 4.9. Cell Culture and Drug Treatment

HUVECs were obtained from Shanghai Bogoo Biotechnology (Shanghai, China). Cells were incubated in DMEM/F12 containing 5 mM D-glucose at 37.0 °C in a humidified atmosphere with 5% CO_2_ in air until 80% confluence. The cells were exposed to different conditions. Medium was replaced every 2–3 days and 24 h before the end of the experiment. All reagents were purchased from Sigma-Aldrich (St. Louis, MO, USA) unless otherwise specified.

### 4.10. Transfection of miR-146a Antagomir

HUVECs were under normal (5 mM D-glucose) and hyperglycemic (25 mM D-glucose) conditions. Concurrent with the creation of cellular hyperglycemia, the HUVECs were transfected in parallel with 20 nM of hsa-miR-146a antagomir or scramble (RiboBio, Guangzhou, China) using HiPerFect reagent (RiboBio), according to the manufacturer’s protocol. After transfection (24 h), the medium was replaced with fresh incubation medium contained fetal bovine serum (FBS). Cells were harvested and subjected to C66 (5 µM) treatments after transfection. The control cells were treated with 0.5% dimethyl sulfoxide (DMSO; Sigma-Aldrich). After incubation for 72 h, cells were harvested for western blot. Transfections were performed with lipofectimine^TM^ 2000 reagents (Invitrogen, Carlsbad, CA, USA). 

### 4.11. Statistical Analysis

All data are presented as means ± S.D. of at least three independent experiments. Comparisons between multiple groups were performed by one-way analysis of variance (ANOVA) with the post-hoc Student–Newman–Keuls test. Values of *p* < 0.05 were considered statistically significant. All statistical analyses were performed with Graph Pad Prism 5.0.

## Figures and Tables

**Table 1:** Primers used for real-time quantitative PCR analysis.

Genes	Sequence (5′-3′)
TNF-α	F: CTCGAACCCCGAGTGACAA
	R: CCGTCGAGGATGTACCGAAT
IL-6	F: ACTTCCATCCAGTTGCCTTCTTGG
	R:TTAAGCCTCCGACTTGTGAAGTGG
IL-8	F: GCTCCTGCTGGCTGTCCTTA
	R: AGCCTTCACCCATGGAGCAT
IRAK1	F: AGGATCAGCTCCACCTTCAGACC
	R: TCGGTGTCCACAGCCAGTCG
GAPDH	F: AGGTCGGTGTGAACGGATTTG
	R: TGTAGACCATGTAGTTGAGGTCA
U6	F: CTCGCTTCGGCAGCACA
mmu-miR-146a	F: CGCGTGAGAACTGAATTCCA
Human-miR-146a	F:GCCCTGAGAACTGAATTCCATG
Universal reverse primer	R: GTGCAGGGTCCGAGGT
F: Forward; R: Reverse	
