# Correction: Huang et al. A Synthetic Curcuminoid Analog, (2*E*,6*E*)-2,6-bis(2-(trifluoromethyl)benzylidene)cyclohexanone, Ameliorates Impaired Wound Healing in Streptozotocin-Induced Diabetic Mice by Increasing miR-146a. *Molecules* 2020, *25*, 920

**DOI:** 10.3390/molecules31203579

**Published:** 2026-10-09

**Authors:** Jingjuan Huang, Jia Fu, Bing Liu, Rui Wang, Tianhui You

**Affiliations:** 1 School of Nursing, Guangdong Pharmaceutical University, Guangzhou 510006, China; 18825077178@163.com (J.H.); fjsxyd1012@163.com (J.F.); 2 Guangzhou Key Laboratory of Construction and Application of New Drug Screening Model Systems, Guangdong Pharmaceutical University, Guangzhou 510006, China; liubing520@gdpu.edu.cn; 3 Key Laboratory of New Drug Discovery and Evaluation of Ordinary Universities of Guangdong Province, Guangdong Pharmaceutical University, Guangzhou 510006, China; 4 School of Pharmacy, Guangdong Pharmaceutical University, Guangzhou 510006, China; 5 Guangdong Key Laboratory of Pharmaceutical Bioactive Substances, Guangdong Pharmaceutical University, Guangzhou 510006, China

## Error in Figure

In the original publication [1], there was a mistake in Figure 3 as published. In Figure 3A, the small images in the first line and the large images in the second line were used to reflect two different outcome indicators: reepithelialization and epidermal thickness of skin wounds, respectively. In the figure assembly, the authors misplaced the position of the small images of the DM/C66-H group and the DM/C66-L group, which means that the positions of these two images should be exchanged. The authors have corrected the positions of the two small images. In addition, on the horizontal axis of Figure 3B, the authors mistakenly wrote the mark of the fifth group of mice as “C66/Curcumin”, and the correct group mark should be “DM/Curcumin”. The corrected Figure 3 appears below. The authors state that the scientific conclusions are unaffected. This correction was approved by the Academic Editor. The original publication has also been updated.

## Figures and Tables

**Figure 3:**
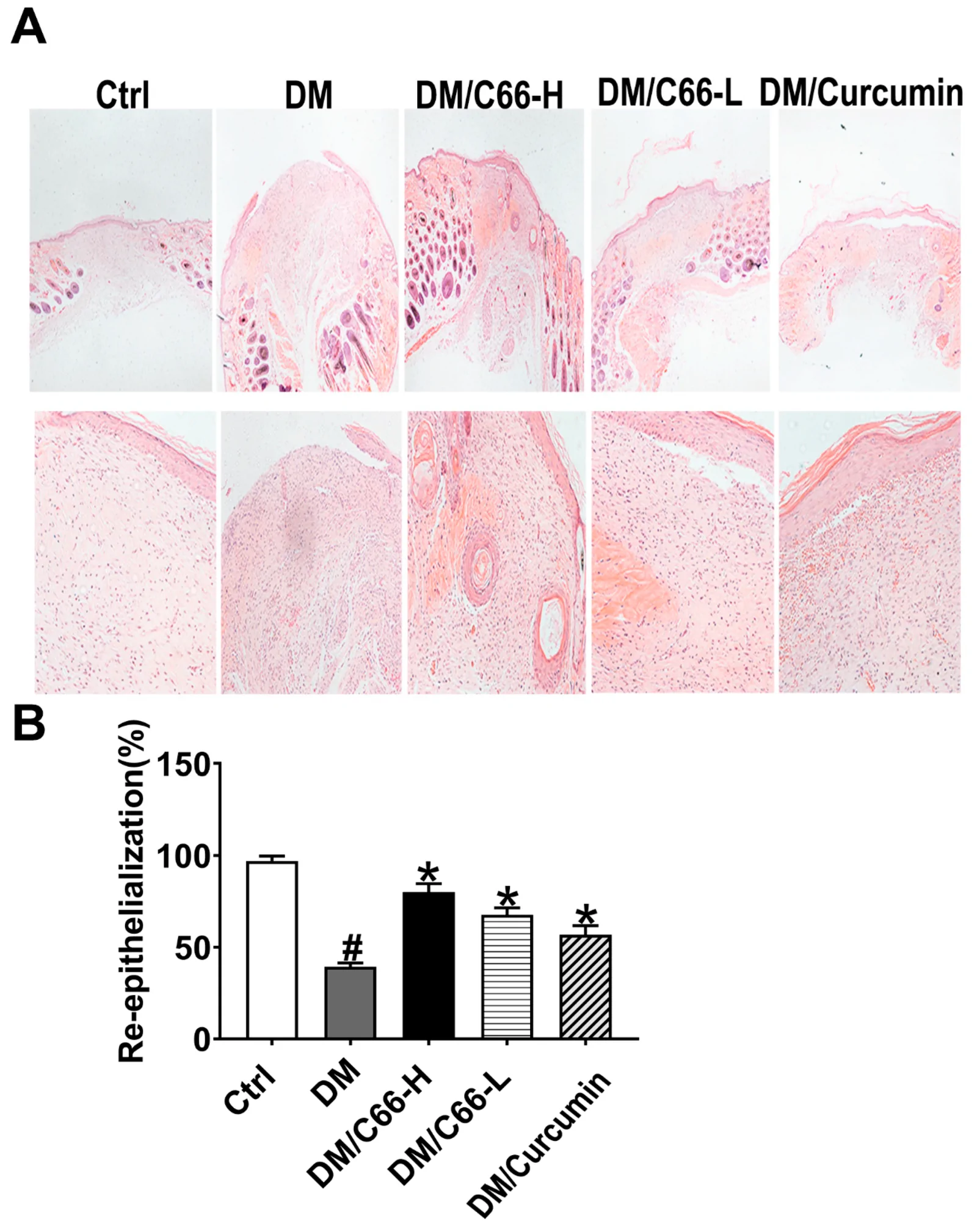
Reepithelialization of skin wounds was accelerated in the C66-treated mice. (**A**) Histological reepithelialization of skin wound in different groups was measured at day 14 after injury (H&E staining, ×100). (**B**) The ratio of reepithelialization was evaluated in different groups. Data are presented as the means ± S.D (*n* = 6 in each group). * *p* < 0.05 compared to the diabetes group value, # *p* < 0.05 compared to the control value. DM and Ctrl are short for the diabetic and control group, respectively.
